# Graphene-ZnO Thin-Film Heterostructure-Based Efficient UV Photosensors

**DOI:** 10.1021/acsaelm.5c00348

**Published:** 2025-05-19

**Authors:** Ravi K. Biroju, Sanat Nalini Paltasingh, Mihir Ranjan Sahoo, Soumen Dhara, Dipak Maity, Viliam Vretenár, P. K. Giri, Tharangattu N. Narayanan, Saroj Kumar Nayak

**Affiliations:** † Centre for Nanodiagnostics of Materials, Faculty of Materials Science and Technology, 61791 Slovak University of Technology, Vazovova 5, 812 43 Bratislava, Slovakia; ‡ School of Advanced Sciences-Division of Physics, Vellore Institute of Technology, Chennai 600048, Tamil Nadu, India; § School of Basic Sciences, 231530 Indian Institute of Technology, Bhubaneswar 752050, India; ∥ School of Applied Sciences, 90940 Kalinga Institute of Industrial Technology, Bhubaneswar 751024, India; ⊥ Surface Science and Interface Engineering Group, 383027 Tata Institute of Fundamental Research Hyderabad, Sy. No. 36/P, Serilingampally Mandal, Gopanpally Village, Hyderabad 500 107, India; # Centre for Nanotechnology and Department of Physics, 28678 Indian Institute of Technology Guwahati, Guwahati 781039, India

**Keywords:** UV photosensors, CVD graphene, ZnO thin film, photoconductivity, photoresponsivity

## Abstract

Graphene-based ZnO thin-film hybrids (GR-ZnO) have shown interesting properties for electronic and optoelectronic applications, such as enhanced UV photodetection and photocatalysis. The interaction and explicit role of large-area single-layer chemical vapor deposition (CVD)-grown graphene in the improved photophysical properties in such a kind of GR-ZnO hybrids have not been well-understood in recent reports. In the present work, we fabricated a photosensor made with large-area monolayer CVD GR-ZnO thin-film hybrids, which showed improved UV photodetection with high values of UV sensitivity and responsivity compared to bare ZnO films. The GR-ZnO thin-film hybrid photosensors demonstrated about a 20 time improvement in photoresponsivity (9.87 × 10^3^ A/W) compared to the bare ZnO thin film (4.93 × 10^2^ A/W). We investigated the origin of the high photosensitivity of GR-ZnO, and it is explained based on a comparatively large absorption coefficient, enhancement of the number of photogenerated carriers, and a reduction of the recombination rates of these carriers based on density functional theory (DFT) calculations. The high mobility of the graphene layer provides an efficient and faster charge transfer pathway for photogenerated carriers at the interface between ZnO and the graphene layers.

## Graphical abstract

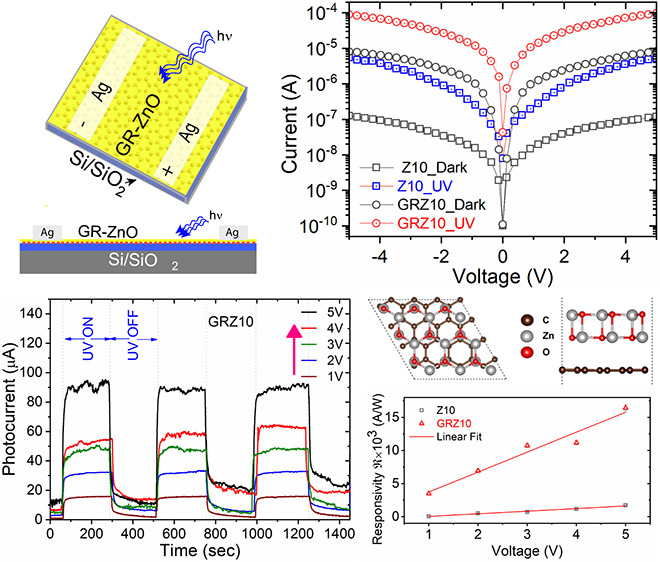

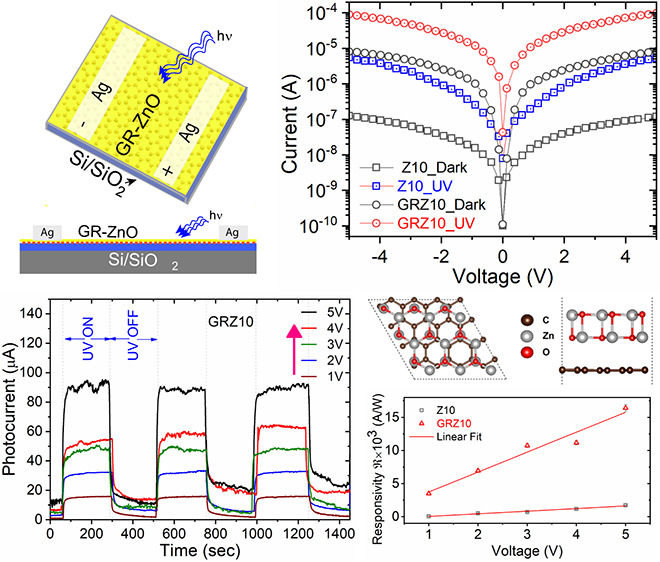

## 1 Introduction

In recent years, graphene-based semiconductor heterostructures with other two-dimensional (2D), one-dimensional (1D), and zero-dimensional (0D) semiconductors have attracted a lot of attention due to their potential applications in photodetection,^1–4^ photovoltaics,^5,6^ photocatalysis,^7^ nanogenerators,^8^ sensors,^9^ and efficient energy devices^10–13^ . Due to the compatibility of graphene with conventional thin-film-based microelectronics, a graphene-based heterostructure with semiconductors is an excellent choice for the future optoelectronic industry.^14,15^ Among all of these, ZnO-graphene hybrid nanostructures are particularly suitable because of their unique photophysical properties of ZnO and graphene and their compatibility with the growth conditions of hybrids. Graphene-based ZnO thin-film hybrids (GR-ZnO) combine the advantages of ZnO (a wide direct band gap, semiconductor, large exciton binding energy, biocompatible, and so forth) and graphene (flexible, a highly conductive layer, environment-friendly, and so forth), which provides a unique platform for applications as photosensors^8–10^ and gas sensors.^16–18^

Under light illumination, the effective separation of photogenerated electron–hole pairs by the local electric field suppressed carrier recombination rates and increased carrier lifetime, leading to an increased free carrier density. In turn, this can reduce the Schottky barrier between graphene and the ZnO thin film, facilitating the transport and collection of photocarriers. As a result, a high *I*_light_/*I*_dark_ can be achieved, much higher than bare ZnO thin films or ZnO nanostructures. Photodiodes based on GR-ZnO heterostructures usually exploit the Schottky junction formed between graphene and ZnO.^17^ The formed built-in potential can separate photogenerated electron–hole pairs at zero-bias conditions, potentially reducing the dark current and thus bringing about an improved specific detectivity.^19^ In addition, under moderate reverse bias, the separation efficiency of photocarriers will be improved as the built-in electric field is further strengthened, resulting in a response much faster than the bare ZnO photodetector.^19^ Incorporating ultrathin semiconductor 2D films such as a ZnO seed layer could enhance the separation of photocarriers by built-in electric fields formed at the GR-ZnO and ZnO-SiO_2_ interfaces. In addition, the ZnO thin film can also serve as an antireflection layer to trap incident light and enhance optical absorption. On the other hand, understanding the interfacial physical phenomena between graphene and ZnO thin films, before fabricating the heterostructures and their devices such as nanowires, nanorods, nanowalls, and quantum dots, is very important.^19,20^ The devices exhibit enhanced photo responsivity for these advantages, especially near-UV to the visible region. Moreover, the response speed can also be greatly reduced by an order of magnitude, with a rise/fall time as low as 280/540 μs.

With the excellent electrical, mechanical, and thermal characteristics of graphene layers, growing ZnO nanostructures and thin films on graphene would enable their novel physical properties to be exploited in a diverse range of sophisticated device applications.^16^ Therefore, several graphene–semiconductor nanostructure hybrids have been successfully synthesized that show elegant combinations of properties not found in the individual components.^17,20^ Recently, Xu et al. developed a metal–semiconductor–metal photosensor using hydrothermally grown ZnO NWs and observed the existence of surface plasmon resonance arising from the underlying graphene layer, which exhibited a UV to visible light rejection ratio of ∼up to 4 orders.^21^ Furthermore, understanding the host–guest interaction of such graphene-ZnO hybrids will be key for novel applications, with their photophysical properties beyond the current state of the art.

In the present work, we demonstrate the fabrication of a hybrid photosensor using a ZnO thin film on a graphene monolayer and its improved photoelectrical properties. For comparison, a photosensor made with a pristine ZnO thin film on a Si/SiO_2_ substrate was fabricated and its photoresponse behavior was investigated. The surface morphology and structural quality of the pristine ZnO and ZnO-graphene thin films were assessed by employing XRD, AFM, FESEM, high-angle annular dark-field scanning transmission electron microscopy (HAADF-STEM) imaging, and Raman spectroscopy. Furthermore, from DFT calculations, we found the formation energy of the GR-ZnO heterostructure ‘–1.74 eV’. This lower negative formation energy with 3.12 A ° interlayer spacing is a clear signature of van der Waals (vdW) interaction between the graphene and ZnO thin film by forming a vdW heterostructure. Here, the substantial increment in electron conductivity between graphene and ZnO may be due to the charge redistribution within the graphene layer, and a large potential drop of 13.8 eV leads to the enhanced UV photoresponse in the GR-ZnO heterostructure photosensor. The improvement observed in the photosensing characteristics of the GR-ZnO photosensor is thoroughly investigated.

## 2 Experimental Section

### 2.1 Fabrication of Graphene Using CVD

Single- and few-layer graphene samples were grown by a thermal chemical vapor deposition (CVD) technique and transferred onto Si/SiO_2_, quartz, and TEM quantifoil grids by the conventional wet transfer method. Full details of the controlled CVD growth of graphene were reported elsewhere.^18,20^ The as-transferred graphene was characterized using HAADF-STEM, and the corresponding low-angle annular dark-field (ADF) of the transferred monolayer graphene and ABSF-filtered STEM images are shown in Figure S1a,b.

### 2.2 Deposition of High-Quality ZnO Thin Film on Graphene

A thin, high-quality ZnO film of thickness ∼10 nm was deposited using RF magnetron sputtering on Si/SiO_2_/graphene substrates and TEM quantifoil grids (sample code: GRZ10). The thickness of the ZnO plays a vital role in determining photosensor performance by influencing optical absorption, which leads to an increase in photocurrent. The thickness of ZnO was chosen 10 nm to expect a higher photocurrent due to better current transfer to the underlying graphene layer. It should be noted that the increased thickness of ZnO may introduce more grain boundaries and surface roughness, which may be ineffective in understanding the graphene and ZnO interface. The effect of ZnO thickness was reported elsewhere.^29^ Graphene showed a significant Raman signal by showing the G, D, and 2D bands from the GRZ10 hybrid film, which was further investigated for UV photosensor fabrication in the present study. A high-purity ZnO sputter target (99.999%, Kurt J Lesker, USA) was used as a source for ZnO grain growth. Initially, the chamber was evacuated to a base pressure of 6.7 × 10^–6^ mbar, and during the sputtering, it was maintained at 1 × 10^–2^ mbar. The RF power was kept at 100 W. The substrate was heated to 200 °C for better crystalline quality and uniformity of the ZnO grains. The deposition time was kept for 15 min. For comparison, an identical ZnO thin film on a Si/SiO_2_ substrate was also grown (sample code: Z10) during the deposition of ZnO on graphene. Both samples were postprocessed by rapid thermal annealing (RTA) treatment at 600 °C in an Ar gas ambient (flow rate of 200 standard cubic centimeters (sccm)) atmosphere for 3 min using a commercial RTA system (MILA3000, Ulvac, Japan) in order to further improve the crystalline quality as well as for the removal of excess oxygen traps on the ZnO thin film on various substrates. Improvements in the crystallinity of ZnO grains and their phases were confirmed by X-ray diffraction (XRD) and X-ray photoelectron spectroscopy (XPS) analyses (discussed later).

### 2.3 Characterization

The surface morphology, topography, and crystallinity of the ZnO thin film on graphene were characterized by FESEM (Sigma, Zeiss), AFM (5500, Agilent Technologies), XRD (Rigaku RINT 2500 TRAX- III, Cu Kα X-ray radiation), and Raman spectroscopy (LabRam HR, Horiba) techniques. XPS measurements were carried out with a fully automated XPS microprobe (PHI-*X*tool, Ulvac-Phi, Japan) using an Al Kα X-ray beam (1486.7 eV). The carbon 1s spectrum was used to calibrate the XPS spectra recorded for various samples. For electrical measurements, the contact pads were made using the thermal evaporation technique on top of the active semiconducting layer, and the thickness of the ‘Ag’ layer was 100 nm. The devices were lifted off using acetone, followed by mild sonication. The area of the device was measured as ∼100 μm × 10 μm. Photoconductivity (PC) and photoresponse measurements were performed with a microprobe station (ECOPIA EPS-500) connected to a source meter (Keithely 2400) for current–voltage (*I*–*V*) characteristics, and a 300 W xenon lamp was used as the light source to excite the sample. It should be noted that the excitation wavelength of the incident light was chosen by using a manual monochromator (Oriel Instruments, USA). It should be noted that the excitation light spot was tightly focused within the active semiconductor channel using appropriate optical accessories. The current–voltage and transient photocurrent data were recorded by using Lab Tracer 2.0 software.

### 2.4 Computational Method

The first-principles calculations are carried out using density functional theory (DFT), accomplished by the Vienna Ab Initio Simulation Package (VASP).^22,23^ The spin-polarized generalized gradient approximation of Perdew–Burke–Ernzerhof (GGA-PBE+U) is used to describe the exchange-correlation potential. Hubbard parameters are used to treat the 3d orbital electrons of Zn, with a value of *U*_*d*_ = 10 eV, and the 2p orbital electrons of O, with a value *U*_*p*_ = 7 eV, following previous studies.^24,25^ The projector-augmented plane-wave (PAW) pseudopotential approach^26^ is adopted to analyze the interaction between core ions and valence electrons. The plane-wave cutoff energy is fixed to 500 eV for all calculations, and all the geometrical structures are optimized by fully relaxing the atomic positions with a total energy convergence criterion of 1.0 × 10^–05^ eV. For the correction of vdW interactions proposed by Grimme, the DFT-D2 ^27^ method has been implemented, which incorporates the vdW correction for heterogeneous structures. The Brillouin zone is sampled with gamma-centered k-point grids of 4 × 4 × 1 for structure optimization and 7 × 7 × 1 grids for density of state calculations.

## 3 Results and Discussion

### 3.1 Graphene-ZnO Thin-Film Hybrid (GRZ10)

Figure 1a,b shows the morphology and topography of the RTA-treated GRZ10 sample. The morphology images depict the formation of a continuous film with an average grain size of 30 nm. The optical images of graphene (GR) and GRZ10 are shown in the Supporting Information, Figures S2 and S3, respectively. The root-mean-square (RMS) roughness of the GRZ10 film was estimated to be ∼5 nm. The structural characterization of the hybrid film was checked by XRD and showed a typical XRD pattern of crystalline hexagonal ZnO, as shown in Figure 1c. The XRD pattern shows strong peaks at 2θ = 31.72°, 34.34°, 36.06°, and 47.62°, corresponding to the (100), (002), (101), and (102) planes of ZnO, respectively. From the XRD data, it is evident that ZnO grains are mostly single crystalline and have *c*- axis orientation in the (002) plane with a perfect Wurtzite structure. It should be noted that the thin ZnO film (10 nm) was deposited at a substrate temperature of 200 °C. ZnO thin films usually demonstrated weak n-type conductivity due to the presence of oxygen vacancy-related defects or native point defects within the material. The chemical states, evolution of vacancy defects, and elemental composition from the Z10 to GRZ10 samples were investigated from XPS and SEM-EDX elemental mapping data, respectively. Figure 2a–c shows the XPS core-level spectra for the elements of Zn-2p, O-1s, and C-1s, respectively. The resultant Zn 2p_1/2_ and Zn 2p_3/2_ core levels are located at binding energies of 1021.7 and 1044.6 eV, respectively, corresponding to stoichiometric of pure hexagonal wurtzite ZnO.^18^ The O 1s core-level spectra show broad bands with centers at 529.16 and 530.93 eV and 529.37 and 530.49 eV corresponding to the GRZ10 and Z10 samples, respectively. Careful deconvolution to the O 1s core-level peak reveals the signature of lattice oxygen attached to Zn atoms (binding energy: 529.16 eV) and the missing oxygen atoms within the ZnO layer (binding energy: 530.93 eV). This is identical to the earlier results on ZnO/graphene hybrids reported by Bae et al.^18^ Furthermore, it is observed that the existence of the bottom graphene layer interacts with the ZnO layer and results in a shift in the corresponding peak position of lattice oxygen. Therefore, the XPS data confirm the existence of surface oxygen vacancies in the ZnO layer, which act as favorable sites for the attachment with the graphene layer. The ZnO layer is expected to serve as an efficient UV absorber, and the attached bottom graphene layer will provide a faster electronic transport pathway.

**Figure 1:**
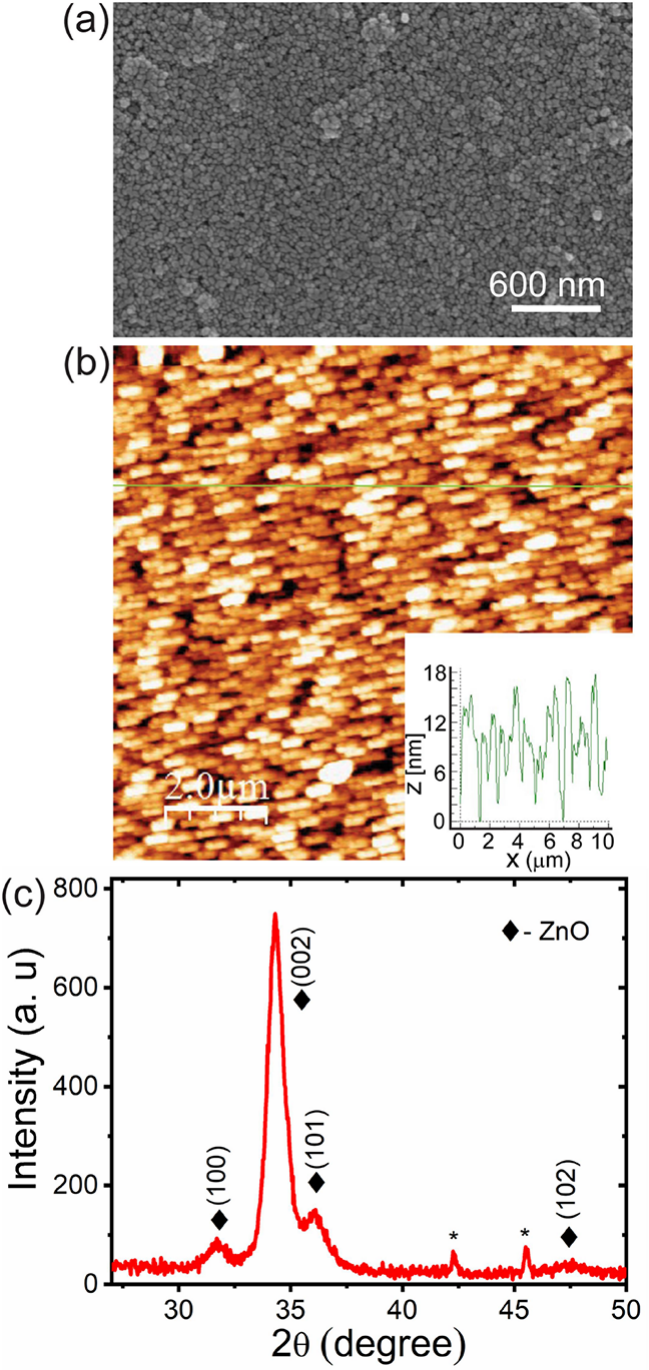
(a) FESEM image of an as-grown ZnO thin film. (b) AFM topography image of a ZnO film and the corresponding line profile of the ZnO grains are shown in the inset. (average size ∼30 nm). (c) XRD pattern of the Z10 thin film after RTA treatment. The prominent peaks correspond to the (100), (101), and (002) planes of wurtzite ZnO.

**Figure 2:**
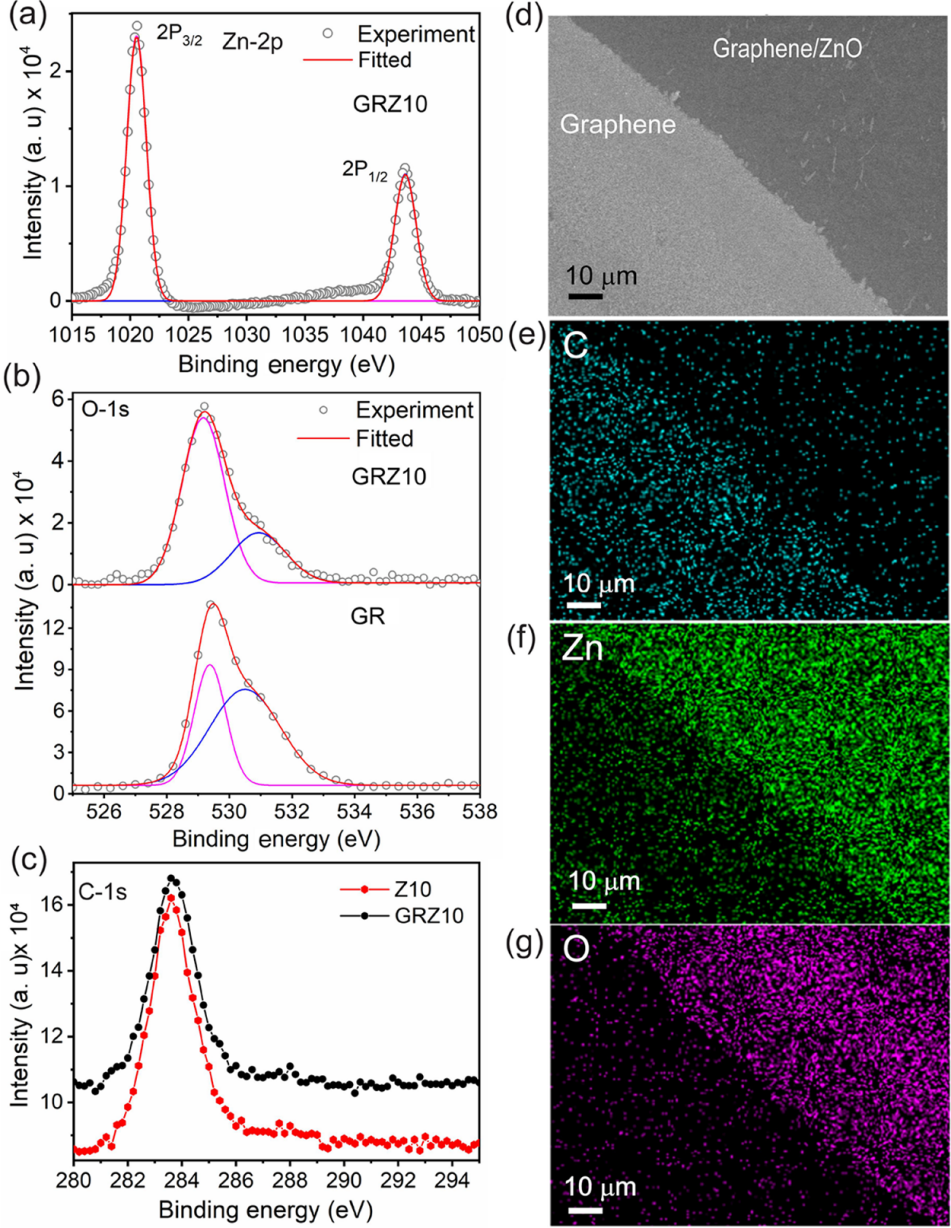
XPS bands for the core-level peaks in graphene-ZnO thin-film hybrids (GRZ10): (a) Zn-(2p_3/2_, 2p_1/2_), (b) O-1s, and (c) C-1s. (d) SEM image of a GR-ZnO masked and unmasked region and corresponding elemental EDS mappings of (e) C, (f) Zn, and (g) O.

Further characterization of the structural quality of the ZnO film and ZnO/graphene was performed by Raman scattering measurements. Figure 3a shows the Raman spectra of the GRZ10 photosensor. The Raman spectra of ZnO and pristine monolayer graphene are shown in the Supporting Information in Figure S4. It should be noted that the Raman spectra of pristine graphene show a very sharp G-band [1586.8 cm^–1^], a strong 2D-band [2678.8 cm^–1^], and a weak D-band [1339.4 cm^–1^], which is a signature of the high crystalline nature of monolayer graphene, which is used for ZnO thin-film deposition. Raman measurements were done after device fabrication, where the laser spot was focused on the active channel layer in between the ‘Ag’ metal contacts, as shown in Figure 4b, which will be discussed later. It should be noted that the fabrication of the ‘Ag’ contacts was made by thermal evaporation, as discussed in the previous section. The presence of a strong D-band and broad G and 2D bands reveals that the underlying graphene has nonuniformity and disorder on the surface due to the large-area coverage of the ZnO film. The background in the Raman spectra may be coming from visible photoluminescence (PL) of the ZnO film. We have also performed UV–vis absorption on GRZ10 and Z10 substrates in order to confirm the graphene-induced modulation/improvement on the absorption of light in the UV–visible region. Both GRZ10 and Z10 show a strong UV absorption band at 360 nm, and a broad visible absorption band ranging from 500 to 800 nm is related to oxygen vacancy-related surface trap centers on the ZnO thin film. The strong UV absorption band in both samples is significant for the single-crystalline nature of the ZnO grains, which is consistent with the micro-Raman and XRD results. Interestingly, the absorption in the visible region is significantly lower compared to the bare ZnO thin film. This is due to the reduction in surface oxygen vacancy defects. Here, note that ZnO grains are preferably attached to the graphene layer at surface vacancy sites, leading to a significant decrease in surface defect centers. It is expected that the observed strong UV light absorption in the GRZ10 sample will lead to a superior visible-blind photosensor. The absorption spectra for the Z10 and GRZ10 layers show a strong UV absorption at ∼360 nm^25^ originated by a radiative transition between the conduction band and energy levels situated just below the conduction band. The broad visible absorption from both Z10 and GRZ10 layers is attributed to the V*_o_*/O*_i_*-related defects on the surface of the ZnO thin film.^28,29^

**Figure 3:**
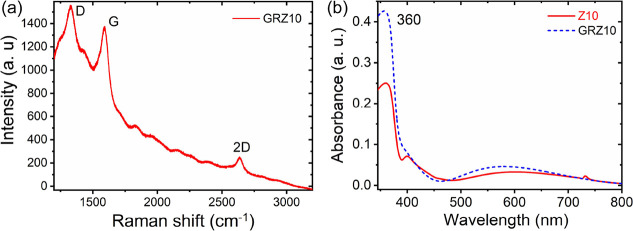
(a) Raman spectra of the GRZ10 photosensor, which is taken at the marked location between the two ‘Ag’ contacts in the GRZ10 device. (b) UV–visible absorption from Z10 and GRZ10 layers coated on quartz substrates shows a strong UV absorption (360 nm) and a broad visible absorption significant for the oxygenated defects on the ZnO surface.

**Figure 4:**
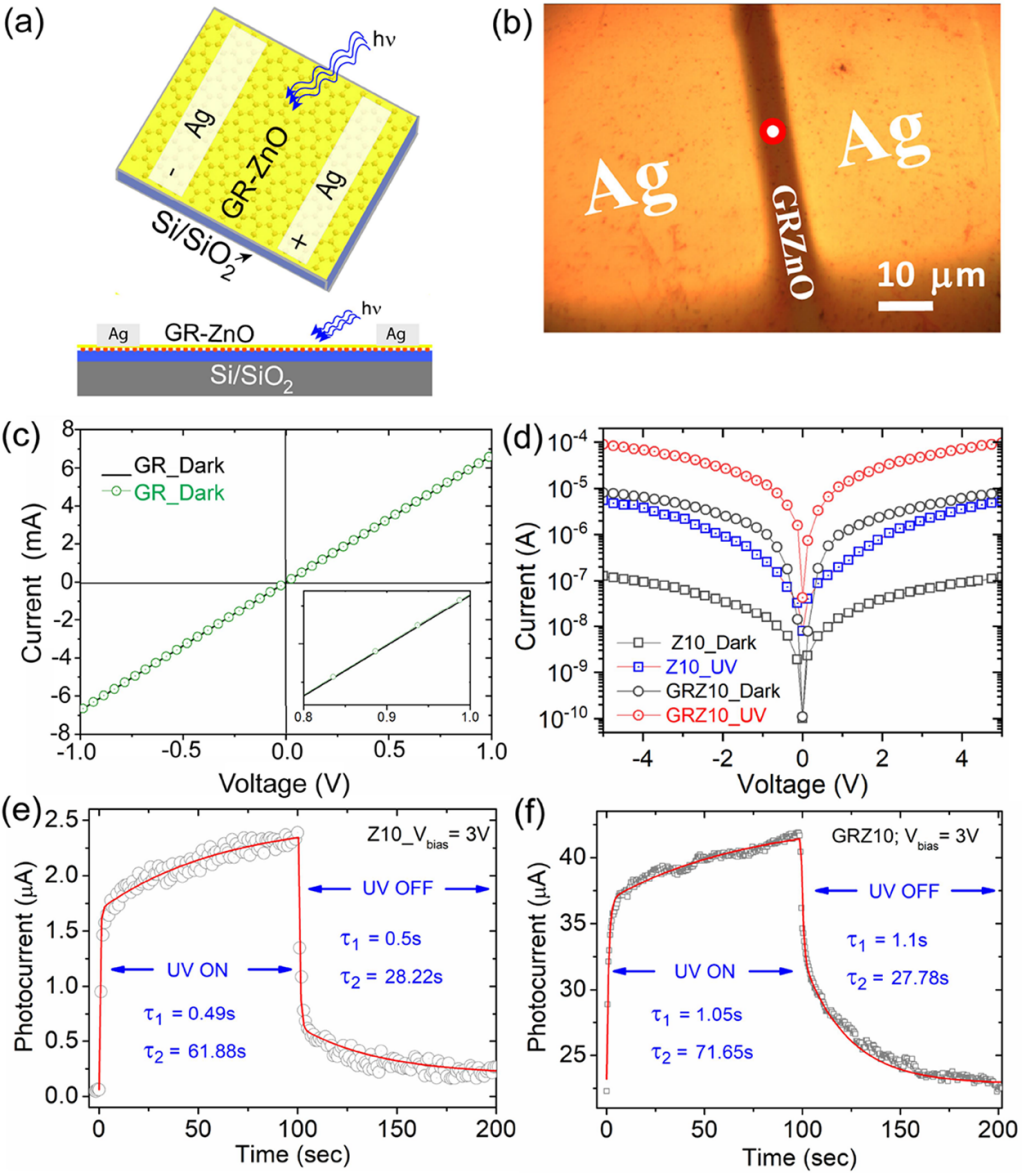
Schematic and optical microscope image of the (a,b) GR-ZnO (GRZ10) photosensor device. (c) Dark and photocurrent *I*–*V* characteristics (excitation wavelength: 375 nm) of CVD-grown graphene, (d) comparison of dark and photocurrent *I*–*V* characteristics of a bare ZnO thin film (Z10) and GRZ10 hybrids. UV photocurrent response as a function of time from the (e) Z10 and (f) GRZ10 thin-film UV photosensors. Photocurrent growth and decay profiles at UV on and off conditions are fitted with the biexponential function. It should be noted that the dotted lines represent experimental data, while the solid red line is a biexponential fitted curve.

### 3.2 Graphene-ZnO UV Photosensor

The conductivity of the ZnO thin film is extremely sensitive to UV light exposure. For a thorough characterization of the fabricated photosensors, we measured the dark and photo *I*–*V* characteristics of both the photosensors made with Z10 and GRZ10 thin films. Figure 4a,b represents the schematic and corresponding optical images of the photosensor device fabricated with GRZ10 layers. It should be noted that a rectangular-shaped active area of the sensor, having a channel width of ∼10 μm, was prepared by depositing the ‘Ag’ layer on the active semiconducting layer employing thermal evaporation and a lift-off process. Figure 4c depicts the dark *I*–*V* characteristic of graphene lying on SiO_2_. It shows a perfectly linear/ohmic nature of the charge transport in the graphene. It is to be noted that the high currents (mA) at a low bias voltage identified in the graphene are significant for the semimetallic nature of the graphene layer. Further dark and photo *I*–*V* curves of GRZ10 and Z10 layers are shown in Figure 4d. Here, the excitation wavelength used for the photoconductivity studies is 375 nm and measured after a light exposure of 30 s. Although the *I*–*V* characteristic of the bare graphene layer demonstrates a perfectly linear nature, *I*–*V* data for the Z10 and GRZ10 photosensors show some deviation from the linear nature. The observed deviation in the *I*–*V* graph is possibly due to the nonuniform potential barrier of ZnO at different bias voltages, which causes nonuniform charge carrier generation. In this case, trap centers present within ZnO, which are commonly observed in nanostructured ZnO, are considered to be responsible. On the other hand, the observed large deviation from linearity in the GRZ10 photosensor is possibly due to the involvement of the ZnO/graphene interface. Here, the charge transport takes place from the ‘Ag’ electrode to ZnO to the graphene layer and returns to the counter ‘Ag’ electrode. Upon UV light illumination, both the photosensors demonstrate significant changes in the photocurrent generation. As expected, the GRZ10 photosensor shows strong photoresponse even at a lower bias, which is clearly visible in Figure 4d. For comparison, the photocurrent generation under the same UV exposure at 375 nm from isolated graphene was checked, and a negligible difference with its dark *I*–*V* counterpart was observed (Figure 4c). Figure 4e,f depicts the transient UV photoresponse for the Z10 and GRZ10 photosensors at a fixed bias voltage of 3 V. Upon UV excitation at 375 nm, the photocurrent increases exponentially and reaches a saturation value after some time. While switching off the UV light, the photocurrent rapidly decays through two different recombination pathways, (1) electron–hole recombination process and (2) recombination through trap centers. To extract all time constants (τ_i_) of growth and decay channels, the transient photosensing data were best fitted with biexponential growth and decay functions as follows.^28,29^ The photocurrent growth equation,

$$I_{\mathrm{ph}}(t)=I_{1}+A_{1}(1-e^{-t/\tau_{1}})-A_{2}e^{-t/\tau_{2}}$$ 1

where *I*_1_, *A*_1_, and *A*_2_ are positive constants. The photocurrent decay equation,

$$I_{\mathrm{ph}}(t)=I_{\mathrm{ph}}(\infty )+A_{3}e^{-t/\tau_{3}}+A_{4}e^{-t/\tau_{4}}$$ 2

where *A*_3_ and *A*_4_ are positive constants, and *I*_ph_(∞)­refers to the photocurrent after an infinitely long time of the decay experiment, which essentially is the dark current. The extracted time constants in Z10 for the photocurrent growth and decay profiles are found to be τ_1_ = 0.49 s; τ_2_ = 61.88 s; τ_3_ = 0.5 s; and τ_4_ = 28.22 s, respectively, while for GRZ10, the values are τ_1_ = 1.05 s; τ_2_ = 71.65 s; τ_3_ = 1.1 s; and τ_4_ = 27.78 s, respectively. For the case of metal oxide, e.g., ZnO, it is attributed that the photoresponse mechanism consists of a two-step process: a rapid process of photogeneration and recombination of electron–hole pairs, and a slow process linked to the surface adsorption and photodesorption of oxygen molecules attaching at the active area of the sensor.^30,31^ During light illumination, photogenerated electrons contribute to the photoconduction process, and photogenerated holes are trapped by adsorbing oxygen (photodesorption process) attached to the surface of ZnO (defect sites). Then, some of the released oxygen atoms recapture available electrons. These two reverse processes lead to a saturated photocurrent, and the sensor reached its maximum photocurrent value. While switching off the light, the dominating electron–hole pair recombination process leads to a faster decay of the photocurrent, and then the oxygen readsorption process brings the photocurrent to the initial value very slowly. We achieved faster response and recovery times in the GRZ10 UV photosensors. Interestingly, the UV photoresponse of GRZ10 is enhanced by a factor of 10 compared to that of Z10, which signifies the high photosensitivity of the ZnO layer in the presence of graphene. In order to understand the nature of UV sensitivity of the GR-ZnO hybrid, we performed cyclic photocurrent measurements on Z10 and GRZ10 photosensors. Figure 5a,b represents the cyclic UV photoresponse characteristics recorded at different bias voltages, 1–5 V. Both the sensors depict uniform repeatability in their photoresponse graph for at least up to three cycles. The responsivity of a photosensor is an important figure of merit which is colligated to the rate of photogenerated charge carriers against the amount of incident optical power at applied bias voltage.^29,32^ The *R*_s_ is defined as

$$R_{s}=\frac{I_{\mathrm{light}}}{P_{0}\times A}$$ 3

where *P*_0_ is the illumination power of incident light (0.405 mW/cm^2^), *A* is the effective exposure area of the device (100 μm × 10 μm), and *I*_light_ is the actual photogenerated current (*I*_ph_ = *I*_light_ – *I*_dark_), where *I*_ph_ is the total photogenerated current, and *I*_dark_ is the dark current at the same bias voltage. The responsivity (*R*_s_) of both Z10 and GRZ10 UV photosensors was calculated as a function of bias voltage, as shown in Figure 5c. The *R*_s_ values for both the photosensors show a gradual increase with the increase in applied bias voltage, which was best fitted with a first-order linear equation. The responsivities of GRZ10 and Z10 photosensors at 3 V bias were found to be 4.93 × 10^2^ A/W and 9.87 × 10^3^ A/W, respectively. This reveals that the responsivity of GRZ10 is higher by a factor of 20 than that of the Z10 layer, which may be due to the Schottky barrier formation between the graphene and ZnO layer coated on it. In conclusion, the cyclic photoresponse measurements reveal that the high mobility of graphene reduces the recombination rate of photogenerated carriers; as a result, the lifetime of the photogenerated carriers was greatly increased.^29,30^ The mechanism of photogenerated charge carrier enhancement is shown in Figure 5d as a type-2 vdW heterostructure band diagram during light excitation and de-excitation. We have also calculated the specific detectivity (D*) of the fabricated photosensor, which is another important figure of merit of the sensor. The specific detectivity is the minimum detectable signal and is a measure of noise in the photodetector. The D* is defined as^33^

$$D^{*}=\frac{R_{s}}{\sqrt{\left(2q\times I_{\mathrm{dark}}\right)}}$$ 4

where *R*_s_ and *I* are the responsivity and dark current at the same bias voltage, and *q* is the electron charge (1.6 × 10^–19^ C). The GRZ10 sensor shows the highest detectivity of ∼9.2 × 10^12^ Jones at a bias of 3 V, which is considered to be a high value compared to conventional photosensors. It should be noted that the sensor made with ZnO only shows detectivity on the order of 10^10^ Jones.

**Figure 5:**
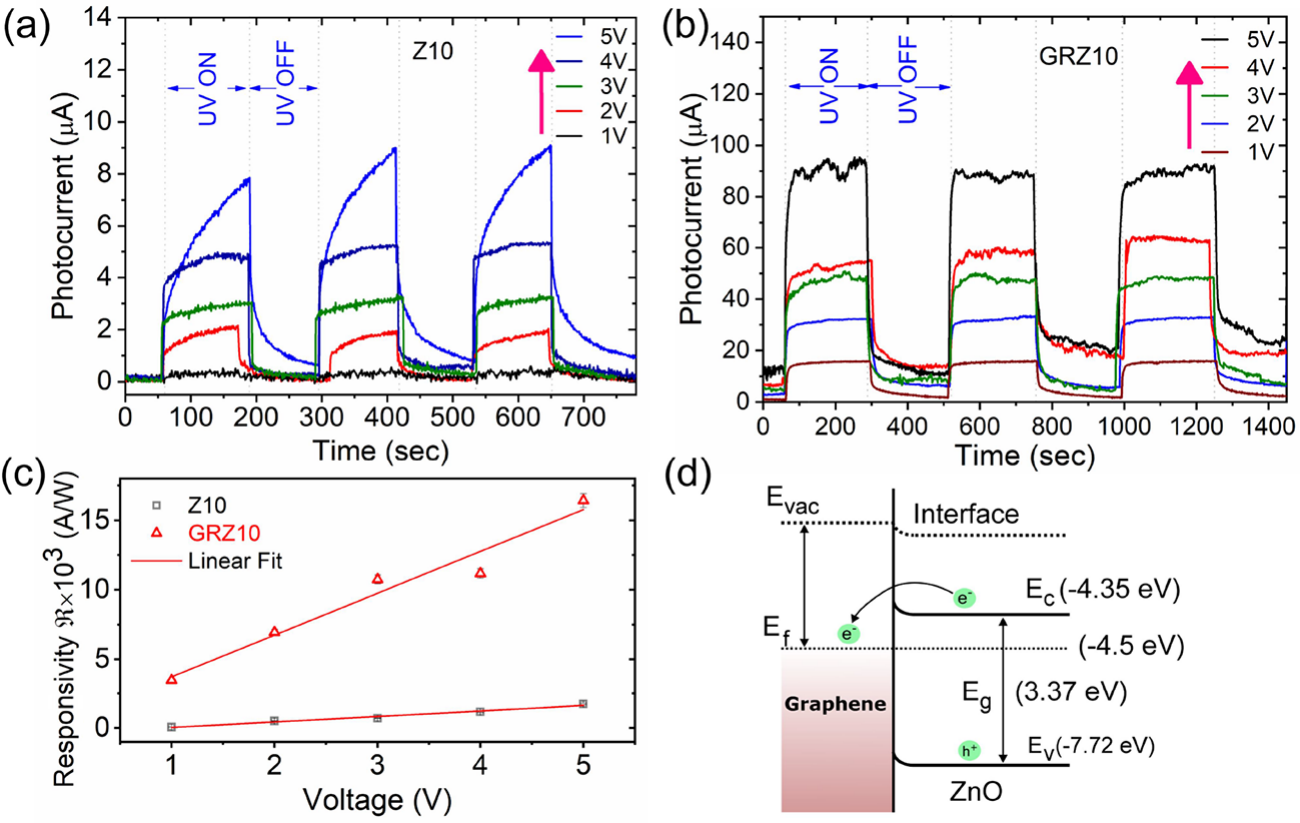
Cyclic photoresponse from ZnO and GR-ZnO thin-film hybrid photosensors: cyclic photoresponse characteristics of (a) Z10 and (b) GRZ10 layers at different bias voltages. (c) Photoresponsivity as a function of bias voltage in Z10 and GRZ10 hybrid photosensors. (d) Band diagram illustrating the graphene and ZnO interface, with charge transfer from ZnO to graphene. Note that the dotted line represents experimental data, and the solid line corresponds to fitted data.

### 3.3 Theoretical Density Functional Theory (DFT)-Based Calculations

In order to understand the interfacial phenomena between graphene and ZnO film, we carried out DFT-based calculations and estimated the electronic as well as optical properties of bare ZnO and the GR-ZnO heterostructure. The wurtzite structure of ZnO exhibits maximal stability under ambient circumstances in which robust covalent bonds interlink the two alternating hexagonal planes of zinc and oxygen lattices.^34^ We used hexagonal wurtzite ZnO to construct the GR-ZnO heterostructure. The lattice parameters of graphene and the ZnO monolayer calculated to set a unit cell are 2.47 and 3.29 Å, which is consistent with previous experimental results and theoretical studies,^35,36^ and to have minimum lattice mismatch we have taken a 3 × 3 × 1 supercell of ZnO and a 4 × 4 × 1 supercell of graphene, which is shown in Figure 6a. After optimization, as shown in Figure 6b a vdW interaction with an equilibrium spacing of 3.12 Å is obtained, which is comparable with other 2D graphene-based heterostructures, such as GR/hBN,^37^ GR/MoS_2_,^38^ and GR/MoSe_2_.^39^ The stability of the heterostructure is verified from the interface formation energy defined by eq 5, as shown below

$$E_{f}=E_{\mathrm{GR}‐\mathrm{ZnO}}-E_{\mathrm{GR}}-E_{\mathrm{ZnO}}$$ 5

where *E*_GR‑ZnO_ is the total energy of the heterostructure, *E*_GR_ is the total energy of graphene, and *E*_ZnO_ is the total energy of the ZnO. The formation energy of the heterostructure is found to be ‘–1.74 eV,’ and this negative formation energy shows that the heterostructure is stable and there exists a vdW interaction between the two layers of graphene and ZnO. To observe the charge distribution nature between the two surfaces graphene and ZnO, the charge density difference (CDD) plot is shown in Figure 6c,d, where the charge accumulation and charge depletion regions are represented by yellow and cyan colors, respectively. The oscillations in the curves of the planar-average-potential plot in Figure 6e reveal that there is an inner-layer charge density rearrangement in each individual, and the oscillation strength is enhanced near the interface. From the charge density difference plot and the corresponding Bader charge analysis, it is found that the graphene and ZnO surfaces do not exchange charges, but the charge redistribution does occur within the graphene layer, leading to the formation of distinct electron–hole puddles at their interfaces, which helps in the increasing mobility of the heterostructure that may substantially boost electron conductivity and develop novel photoconductive and photocatalytic activities. To verify this, the electrostatic potential of the Gr-ZnO heterostructure was determined in the *z*-direction and is shown in Figure 6f, with the vacuum level representing the region outside the surface where the potential remains constant. The work function defined by ϕ = *E*_vacuum_ – *E*_f_ is found to be 4.36 eV, where *E*_vacuum_ represents the electrostatic potential in the vacuum zone, and *E*_f_ represents the Fermi energy. The three humps in the potential plot correspond to the three different atoms C, Zn, and O along the *Z*-axis. The electrostatic potential of C of graphene is deeper than ZnO, and this large potential drop of 13.8 eV suggests a powerful electrostatic field across the interface, which facilitates the mobility of charge carriers across the interface.

**Figure 6:**
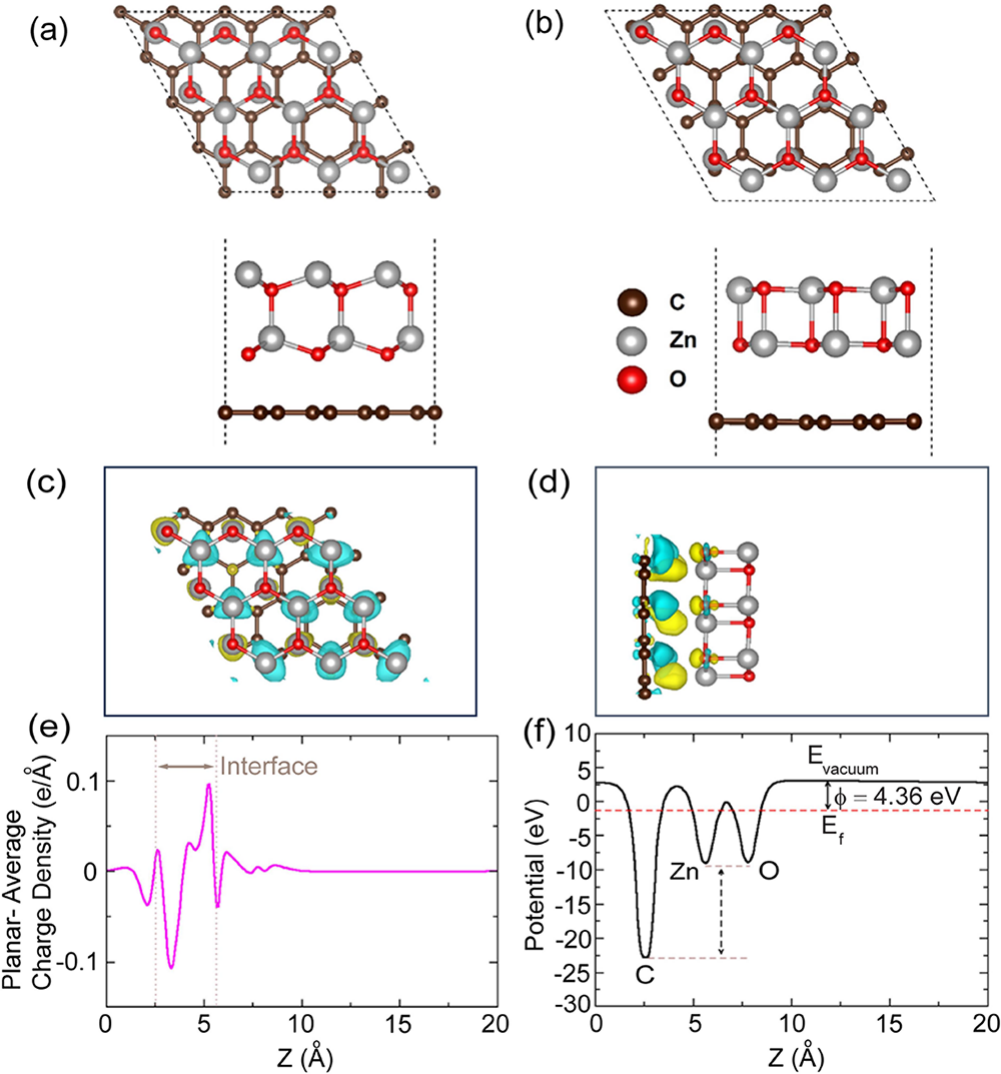
(a) Hexagonal graphene and wurtzite ZnO heterostructure. (b) Optimized GR-ZnO heterostructure. (c) Top view and (d) side view of charge density difference plot of GR-ZnO heterostructure. (e) Planar-average charge density along the *z*-direction, and (f) electrostatic potential of the GR-ZnO heterostructure.

The calculated band diagram and corresponding density of states (DOS) and partial density of states (PDOS) for bare ZnO and Gr-ZnO heterostructures are shown in Figure 7. The monolayer of ZnO was found to have a direct bandgap of 3.07 eV. The computed band gaps using Hubbard U semiempirical corrections indicate both valence band maxima and conduction band minima positioned at the Γ point of the Brillouin zone (BZ). As can be seen in Figure 7b the weak interaction between the two layers in the GR-ZnO vdW heterostructure resulted in a vanishingly small bandgap of 4 meV. This negligible bandgap implies that an electron in the valence band of the heterostructure requires truly low energy (4 meV) to move to the conduction band, which indicates a better mobility characteristic of the heterostructure in comparison to the bare ZnO surface. The Fermi level of the Gr-ZnO heterostructure resides within the induced gap, signifying the absence of charge transfer between graphene and the ZnO monolayer of the GR-ZnO heterostructure, which is also evidenced by their differential charge density (Δρ = ρ_Gr‑ZnO_ – ρ_Gr_ – ρ_ZnO_) illustrated in Figure 6d earlier. By further considering the DOS and PDOS of the heterostructure, as shown in Figure 7c–f, it is observed that the p-orbital of C in graphene dominates the edges of the Dirac cone of the GR-ZnO heterostructure, due to which the bandgap of ZnO reduced to negligible value while forming the vdW heterostructure. According to deformation potential theory,^40^ the effective mass of charge carriers is inversely proportional to the second derivative of the total energy *E*(*k*) and was calculated from the band diagram following eq 6, as shown below:

$$m^{*}=\frac{\hbar^{2}}{\frac{d^{2}E}{dk^{2}}}$$ 6

and for ZnO and GR-ZnO, the effective mass of electrons is found to be 3.2 × 10^–31^ and 0.15 × 10^–31^ kg, respectively. Since the effective mass of charge carriers depends inversely on their mobility, the value of effective mass indicates that the mobility of the charge carriers in the GR-ZnO heterostructure is far greater than the mobility of charge carriers in bare ZnO.

**Figure 7:**
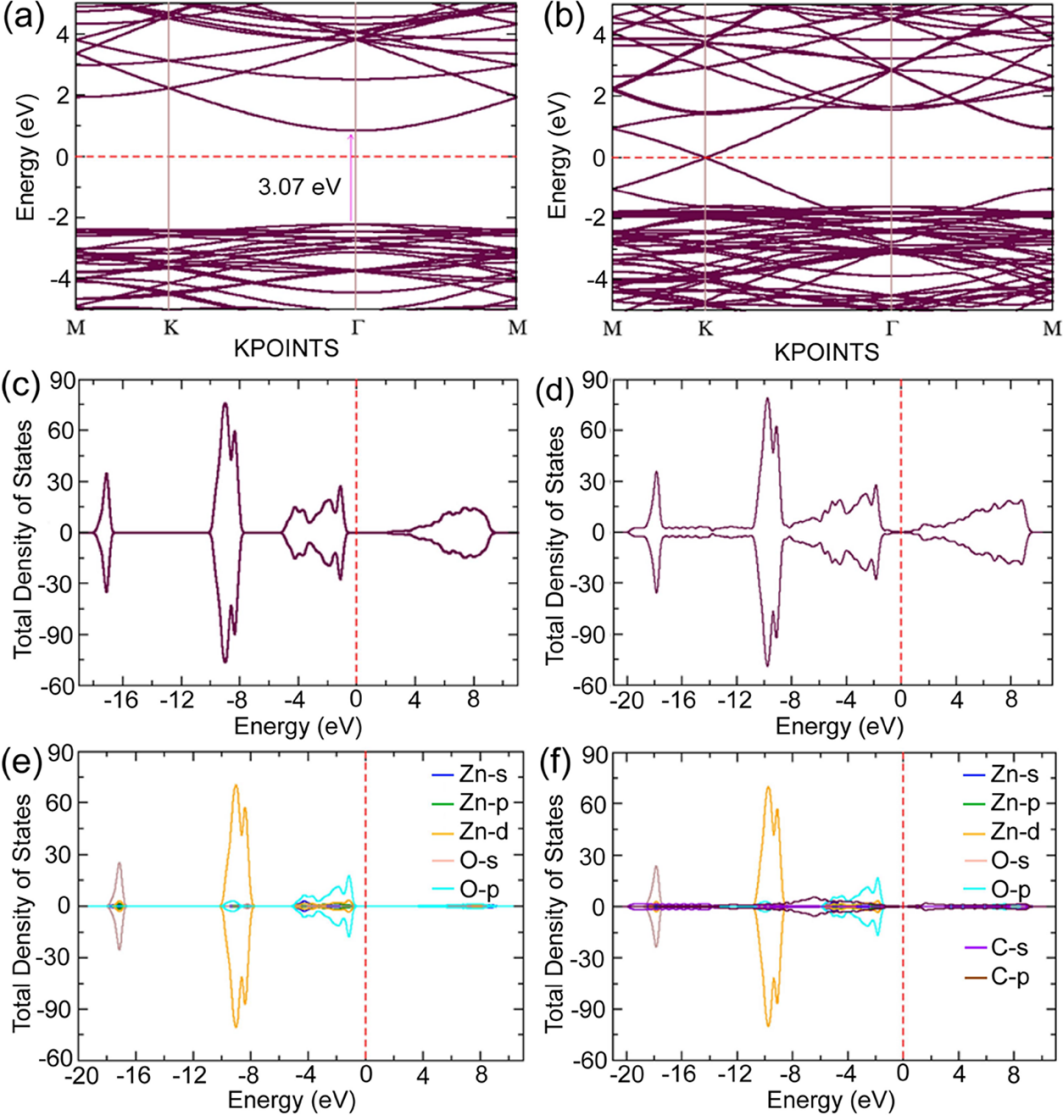
Band diagram of (a) ZnO and (b) GR-ZnO heterostructure. Total density of states of (c) ZnO, and (d) GR-ZnO heterostructure. Projected density of states in (e) ZnO and (f) GR-ZnO heterostructure.

Furthermore, to grasp the material’s behavior toward light excitation, the absorption coefficient property of both bare ZnO and the GR-ZnO heterostructure is obtained from DFT calculations, which indicates the material’s capacity to trap photons with specific energies and is shown in Figure 8. The graphic makes it obvious that the GR-ZnO heterostructure has a strong interaction with radiation as it has a greater absorption coefficient rate than the bare ZnO in all the energy range. For bare ZnO, the absorption begins when the photon energy is near 2.9 eV, which is close to the previously calculated bandgap of 3.07 eV, while for the GR-ZnO heterostructure, the absorption starts just after 0.5 eV, and it shows sharp peaks in the energy range 1–3 eV, which falls in the spectrum of the visible region, where there is negligible absorption for bare ZnO. This indicates that in comparison to bare ZnO, the GR-ZnO heterostructure could respond with better efficiency to the radiation in the UV region, whose spectrum falls in the energy range of 3–12 eV, and even in the visible energy range radiation where bare ZnO fails to respond, which confirms the better photosensing ability of GR-ZnO as seen from experimental results.

**Figure 8:**
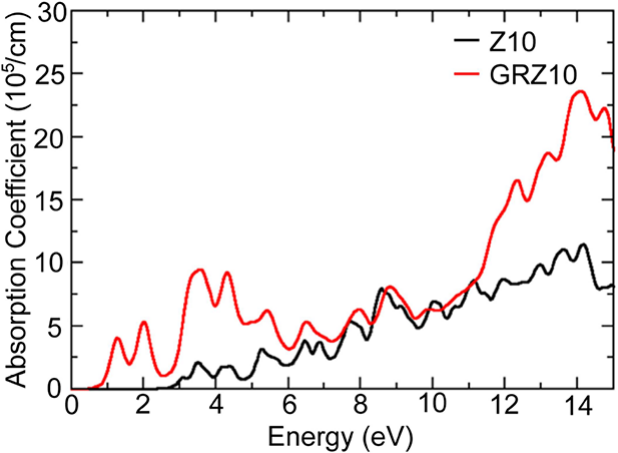
Absorption coefficient of bare ZnO and GR-ZnO calculated using DFT.

## 4 Conclusions

In summary, an efficient UV photosensor based on a CVD-grown graphene–ZnO thin-film hybrid is fabricated and presents its optoelectronic properties. The photoresponse from the GR-ZnO hybrid photosensor showed an enormous responsivity (4.93 × 10^3^ A/Watt), which is a higher factor of 20 in comparison with the bare ZnO thin film on the Si/SiO_2_ substrate. Furthermore, from DFT calculations, it has been seen that the charge carrier mobility in GR-ZnO is higher than that of bare ZnO, which is verified by reduction in the bandgap and effective mass calculations. The electrostatic potential of GR-ZnO, having an inbuilt potential of 13.8 eV, reveals a reduction in charge recombination in the interface of graphene and ZnO, which is beneficial for individual electron capture in device-based applications. Finally, the absorption coefficient plot of the DFT study shows a higher absorption capacity of GR-ZnO and better responsivity to light in the UV region. This kind of novel GR-ZnO hybrid will open new paradigms of novel 2D heterostructures and their optoelectronic properties for advanced electronic circuits in current device applications.

## Supplementary Material



## Notes

Low-angle annular dark-field (LAADF) and corresponding ABSF-filtered scanning transmission electron microscope (STEM) image along with an FFT image of the selective area electron diffraction pattern from single-layer graphene transferred onto a TEM quantifoil grid; optical microscope and SEM images of graphene and graphene-ZnO (GR-ZnO) thin-film hybrid fabricated on Si/SiO_2_ substrate; Raman spectra of a bare ZnO and GR-ZnO thin-film hybrid scanned at 3 different locations on Si/SiO_2_ substrate; and description of photoresponsivity calculations in bare ZnO (Z10) GR-ZnO (GRZ10) sample (DOCX)

## ∇

Institute of Theoretical and Computational Physics, Graz University of Technology, 8010 Graz, Austria
