# Supplementary material for: Graphene-ZnO Thin-Film Heterostructure-Based Efficient UV Photosensors
Source: ACS Appl Electron Mater. 2025 May 19;7(11):4888–97. doi: 10.1021/acsaelm.5c00348 (PMC12160053; doi:10.1021/acsaelm.5c00348)
Supplement: Supplementary file 1 [file el5c00348_si_001.docx]

**Electronic Supporting Information**

**Graphene-ZnO Thin Film Heterostructures based Efficient UV Photosensors**

Ravi Kumar Biroju^1, 2^^[[1]](#footnote-1)^, Sanat Nalini Paltasingh^3^, Mihir Ranjan Sahoo^3#^, Soumen Dhara^4^, Dipak Maity^5^, Viliam Vretenár^1^, Pravat Kumar Giri^6^, Tharangattu Narayanan Narayanan^5^, and Saroj Kumar Nayak^3^

^1^Centre for Nanodiagnostics of Materials, Faculty of Materials Science and Technology, Slovak University of Technology, Vazovova 5, Bratislava, 812 43.

^2^School of Advanced Sciences-Division of Physics, Vellore Institute of Technology Chennai-600048, Tamil Nadu, India.

^3^School of Basic Sciences, Indian Institute of Technology Bhubaneswar, India, 752050.

^4^School of Applied Sciences, Kalinga Institute of Industrial Technology, Bhubaneswar 751024, India.

^5^Surface Science and Interface Engineering Group, Tata Institute of Fundamental Research Hyderabad, Sy. No. 36/P, Serilingampally Mandal, Gopanpally Village, Hyderabad, 500 107, India

^6^Centre for Nanotechnology and Department of Physics, Indian Institute of Technology Guwahati, Guwahati 781039, India.


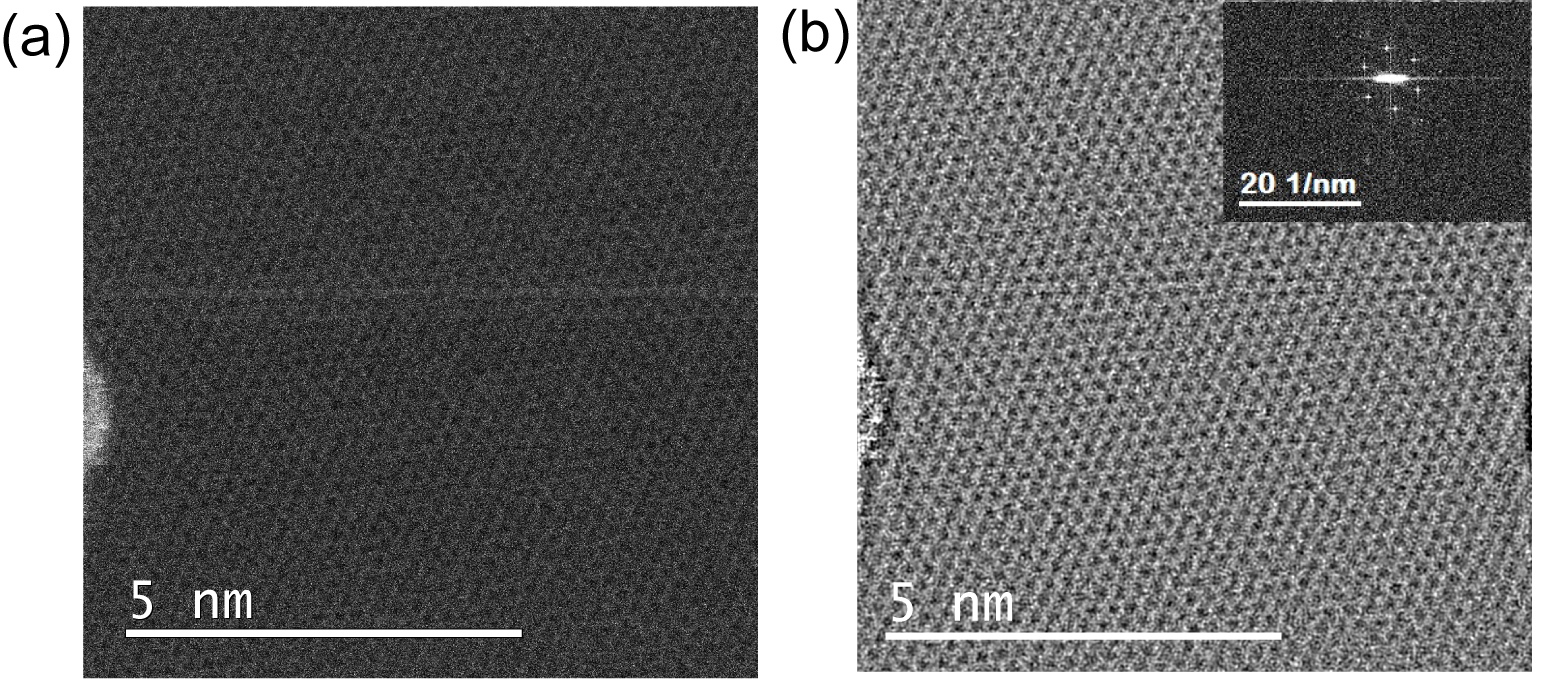


**Figure S1:** (a) Low-angle annular dark field (LAADF) and (b) corresponding ABSF filtered scanning transmission electron microscope image of a single layer graphene transferred onto a quantifoil TEM grid. Note that the inset shows FFT image of the selective area electron diffraction pattern extracted from figure (a).


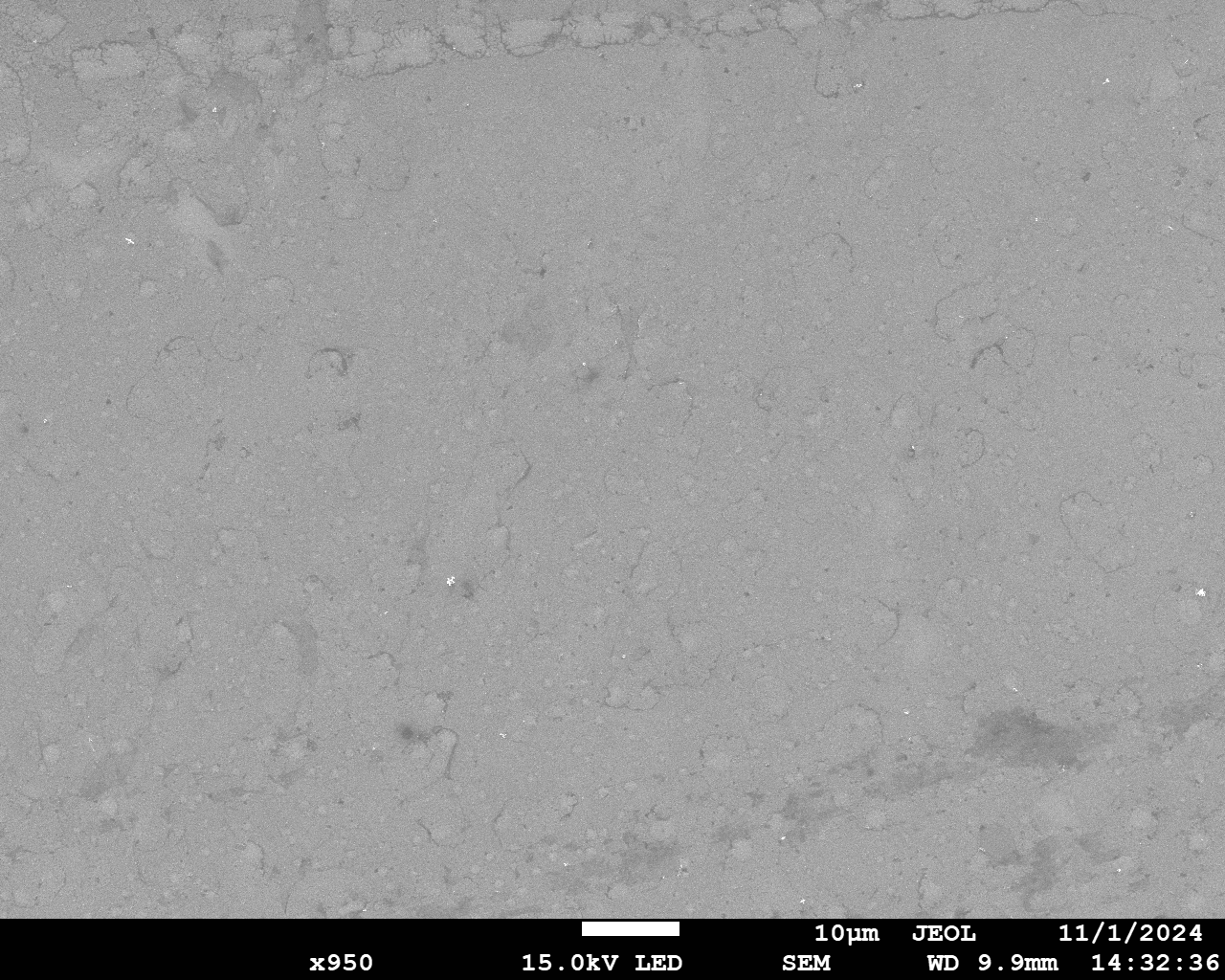

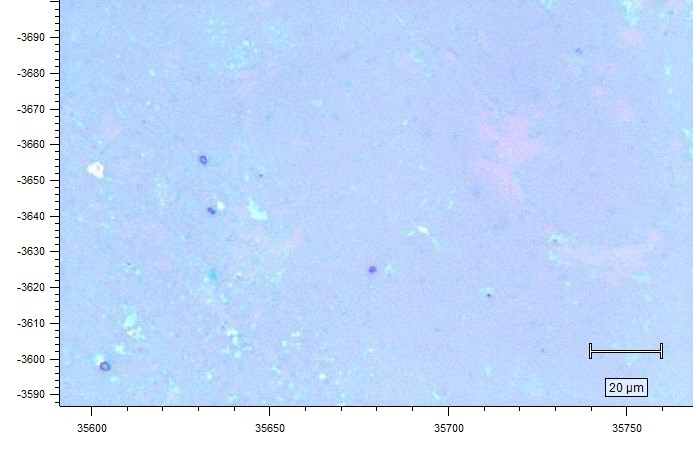


**Figure S2:** Optical image and SEM image of the ~~G~~graphene thin film.


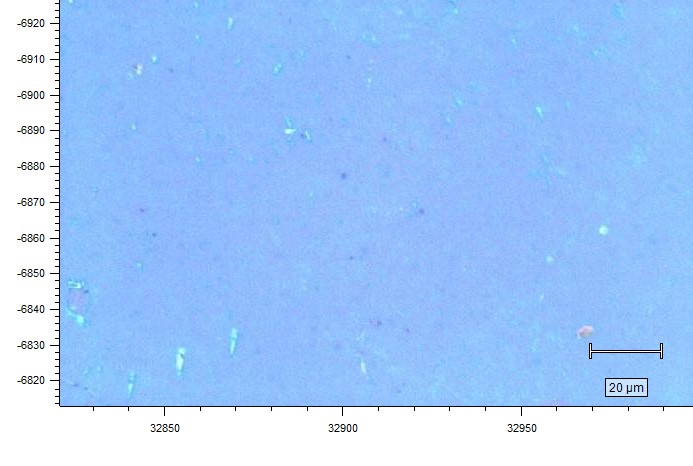

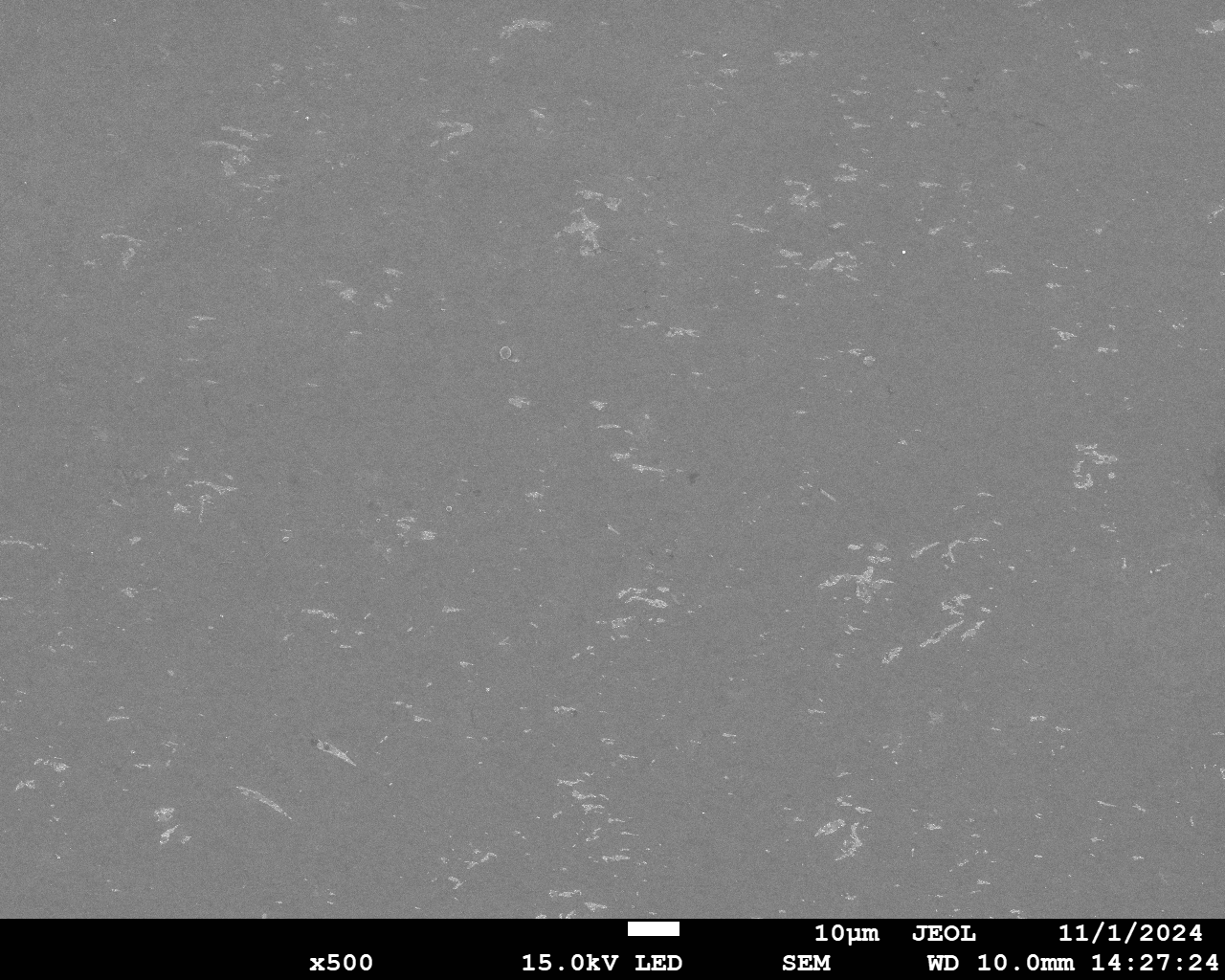


**Figure S3:** Optical image and SEM image of the GR-ZnO thin film hybrid.


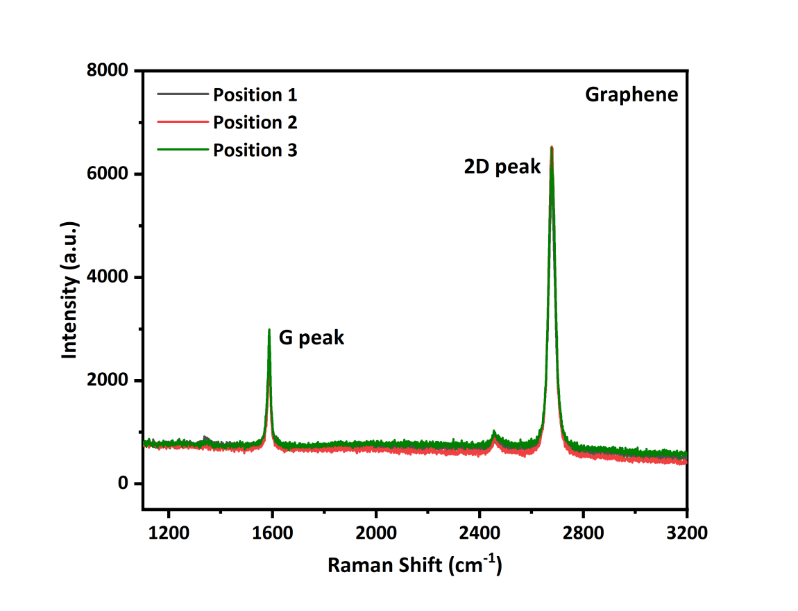

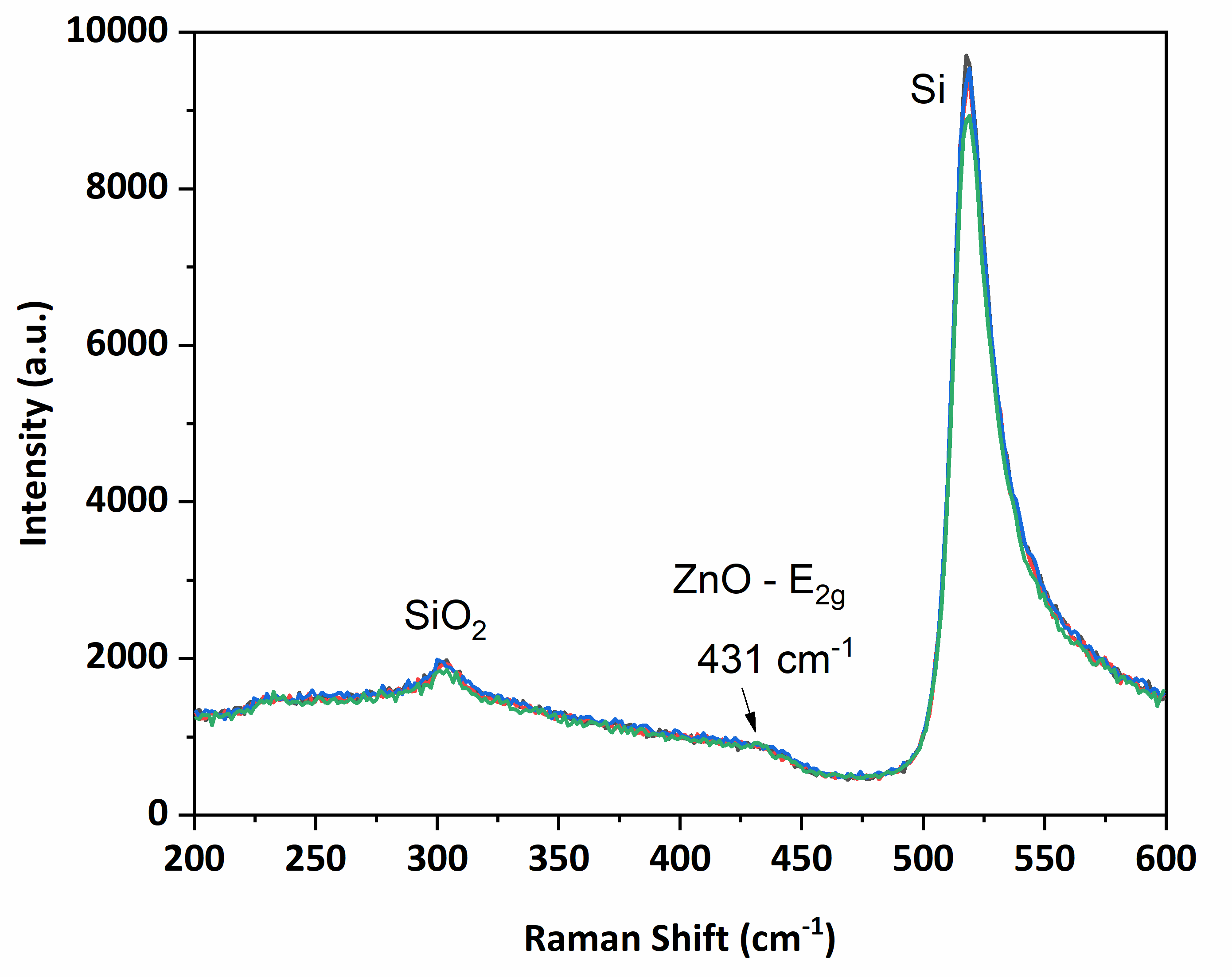


**Figure S4:** Raman spectra of ZnO and pristine graphene.

**SI 1. Calculation of Photoresponsivity in GR-ZnO Hyrbid:**

Responsivity (R) = $\frac{I_{Ph}}{P. A}$

Where A = Geometrical area of the device = 100 μm x 10 μm

P = Power density of an illuminated light = 0.405 mw/cm^2^

I_Ph_ = 2 μA for Z10 sample, bias voltage V = 3V

40 μA for GR-Z10 sample, bias voltage V = 3V

R for Z10 = 4.93 x 10^2^ A/Watt

R for GRZ10 = 9.87 x 10^3^ A/Watt

1. Corresponding author E-mail: Ravi K. Biroju ([ravi.biroju@stuba.sk](mailto:ravi.biroju@stuba.sk); ravikumar. biroju@vit.ac.in)

   ^#^ Institute of Theoretical and Computational Physics, Graz University of Technology, Austria. [↑](#footnote-ref-1)
